# Inflammatory gut–liver crosstalk: mechanisms and therapeutic targets

**DOI:** 10.1038/s12276-026-01810-3

**Published:** 2026-08-06

**Authors:** Atsushi Murao, Monowar Aziz, Ping Wang

**Affiliations:** 1 Center for Immunology and Inflammation, The Feinstein Institutes for Medical Research, 350 Community Drive, Manhasset, NY USA; 2 Departments of Surgery and Molecular Medicine, Zucker School of Medicine at Hofstra/Northwell, 350 Community Drive, Manhasset, NY USA

**Keywords:** Immunology, Cell biology

## Abstract

The gut has a profound influence on the liver through their anatomical connection via the portal vein. During acute inflammation, gut tissue injury leads to increased barrier permeability, allowing the translocation of external contents that can affect hepatic function. Gut–liver crosstalk contributes to the pathophysiology of acute inflammatory disorders, such as sepsis, intestinal ischaemia–reperfusion, hepatitis and drug-induced liver injury. This organ-to-organ crosstalk is mediated by the microbiome, pathogen-associated molecular patterns (PAMPs), damage-associated molecular patterns (DAMPs) and various proinflammatory mediators. Different types of gut and liver resident cells as well as circulating cells also facilitate inflammatory gut–liver crosstalk. These cell types include intestinal epithelial and myeloid cells, Kupffer cells, sinusoidal endothelial cells, hepatic stellate cells, hepatocytes, lymphocytes and neutrophils. PAMPs and DAMPs activate pattern recognition receptors, such as Toll-like receptors, on various cells, leading to proinflammatory signal transduction, including NFκB activation, cytokine and chemokine production, and NETosis. Collectively, these soluble and cellular factors exacerbate acute inflammation and tissue injury via the gut–liver axis, leading to poor outcomes in critically ill patients. Potential therapeutic interventions for this deadly clinical condition include modulation of the microbiome and pharmacological inhibition of proinflammatory mediators and cellular interactions. In this article we review the pathophysiology of inflammatory gut–liver crosstalk and potential therapeutic interventions.

## Gut–Liver Crosstalk Drives Acute Inflammation and Injury

The gut–liver axis plays a crucial role in maintaining homeostasis and is implicated in various diseases. This review explores the complex interactions between the gut and liver, particularly during acute inflammation. The authors highlight the role of gut microorganisms, microenvironment and immune cells in mediating this crosstalk. They describe how gut-derived pathogen-associated molecular patterns and damage-associated molecular patterns activate gut-resident immune cells, which subsequently interact with and activate hepatic cells, thereby exacerbating liver injury. Key findings include the identification of gut-primed neutrophils that migrate to the liver, activating Kupffer cells and causing hepatic damage. This understanding is significant as it underscores the potential of targeting the gut–liver axis therapeutically. Future directions may involve developing strategies to modulate the microbiome and inhibit proinflammatory pathways to mitigate acute liver injury during systemic inflammation.

This summary was initially drafted using artificial intelligence, then revised and fact-checked by the author.

## Introduction

The gut and liver are two of the most vital organs in the body implicated in normal homeostasis as well as in disease pathophysiology^1^. The gut digests and absorbs nutrients while serving as a barrier against luminal microbes and toxins. The liver maintains homeostasis by regulating metabolism and removing toxins and pathogens from the body. The gut can heavily influence the liver via their anatomical connection by the portal circulation^1^. During systemic inflammation, such as gut ischaemia–reperfusion (I/R) or sepsis, there can be compromise of the gut barrier function. This is in part due to reduced blood flow and oxygen supply, local inflammatory mediators and resident-cell activation, which can all contribute to further liver injury^2^. Gut microorganisms and microbial antigens modulate gut and liver homeostasis and dysbiosis, an imbalance in the microbiota, can lead to liver inflammation. Pathogen-associated molecular patterns (PAMPs), such as lipopolysaccharides (LPS), entering through the damaged gut, and damage-associated molecular patterns (DAMPs), such as extracellular cold-inducible RNA-binding protein (eCIRP) and high mobility group box 1 (HMGB1), produced in the inflamed gut, can activate or injure hepatic cells^3,4^. Different types of immune and non-immune cells can be activated in the inflammatory gut and produce proinflammatory mediators that can affect the liver or migrate to the liver to more directly activate hepatic cells, exacerbating tissue injury^5,6^. Gut resident cells (e.g. epithelial cells, macrophages and lymphocytes), liver resident cells (e.g. Kupffer cells and sinusoidal endothelial cells), and circulating neutrophils coordinately facilitate gut–liver crosstalk. Recently, a striking finding from our laboratory has demonstrated that during acute inflammation, neutrophils are primed by gut intraepithelial lymphocytes (IELs) to become a hyperactivated NETotic subpopulation, which migrate to the liver, where they activate Kupffer cells and cause hepatic injury^7^. Therapeutic strategies targeting the gut–liver axis during inflammation involve maintaining the microbiome with probiotics and pharmacologically inhibiting proinflammatory molecules, signalling pathways and cellular interactions.

In this review, we summarize the mechanisms underlying inflammatory gut–liver crosstalk, with a focus on the microbiome, proinflammatory mediators and the roles of different cell types. Although the liver can influence the gut to some extent, our review primarily focuses on the gut-to-liver signalling, which is generally regarded as the predominant direction within this axis. We further discuss the therapeutic approaches targeting gut–liver crosstalk and future perspectives in this area.

## Gut–liver crosstalk in health

Although anatomically distinct, the gut and liver are functionally and metabolically connected through the portal circulation and shared immune and microbial networks^8^. Under healthy conditions, the intestinal epithelium and mucus layer form an effective barrier that restricts microbial translocation. Myeloid cells and lymphocytes sense microbial components in the lamina propria, promoting immune tolerance and maintaining mucosal homeostasis^9^. The liver, positioned downstream of the gut via the portal vein, is the prime detoxification organ in the body and plays a major role in the clearance of gut-derived toxins^10^. Even under healthy conditions, the liver is continuously exposed to low concentrations of gut-derived microbial metabolites and antigens, which contribute to immune conditioning and metabolic regulation^10^. Hepatic immune cells, such as Kupffer cells, and liver sinusoidal endothelial cells interpret these signals to maintain a tolerogenic environment^8^.

## Gut–liver crosstalk in acute inflammatory diseases

During acute inflammatory states, the integrity of the gut–liver axis becomes compromised owing to increased intestinal permeability, translocation and circulation of proinflammatory mediators, and aberrant cellular activity. As a result, the homeostatic relationship between the gut and liver transforms into a pathophysiological one. Conditions such as sepsis, intestinal I/R injury, alcoholic hepatitis, drug-induced liver injury and viral hepatitis demonstrate the interaction between the gut and liver in acute inflammation (Table 1).

**Table 1:** Inflammatory disorders mediated by gut–liver crosstalk.

Disease conditions	Key contributors	Manifestations	Refs.
Sepsis	PAMPs, DAMPs, impaired bacterial clearance, dysbiosis, barrier disruption, hyperactivated neutrophils, Kupffer cell M1 polarization	Liver injury, multiorgan dysfunction, high mortality	^2,4,7,11–15^
Gut I/R	Hypoxia-induced DAMPs, PAMP flux, NF-κB, Nrf2/ARE, AMPK/SIRT1, Kupffer cell M1 polarization	Liver injury (↑ AST/ALT), systemic inflammation, high mortality	^16–19^
Alcoholic hepatitis	Dysbiosis, barrier dysfunction, endotoxaemia, hepatocyte injury	Steatohepatitis, liver failure, high mortality	^3,20,21^
Drug-induced liver injury	Microbiome disruption, barrier compromise, PRR activation	Hepatic inflammation, acute liver failure	^22–27^
Viral hepatitis	Gut viral exposure, dysbiosis, barrier disruption, PAMP flux	Acute hepatitis, acute-on-chronic liver failure	^28–30^

*DAMP* damage-associated molecular pattern, *I/R* ischaemia–reperfusion, *PAMP* pathogen-associated molecular pattern, *PRR* pattern recognition receptor.

### Sepsis

**Fig. 1:**
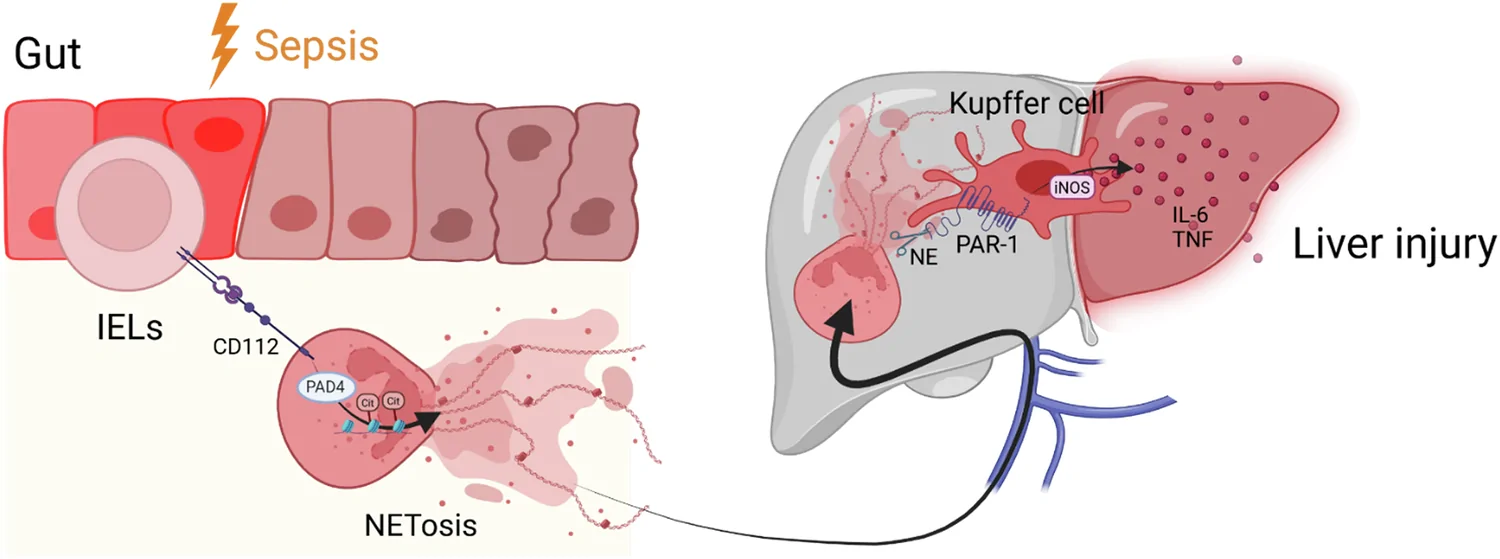
Gut-primed neutrophils facilitate proinflammatory gut–liver crosstalk in sepsis. During sepsis, neutrophils interact with gut intraepithelial lymphocytes (IELs) via CD112, leading to NETosis through peptidylarginine deiminase 4 (PAD4)-mediated histone citrullination. These neutrophils migrate to the liver, where they activate Kupffer cells via protease-activated receptor-1 (PAR-1) by NET-contained proteases, such as neutrophil elastase (NE). As a result, Kupffer cells adopt an iNOS-expressing M1 phenotype and produce proinflammatory cytokines, such as IL-6 and TNF, to cause liver injury^7^. Reproduced with publisher’s permission.

Sepsis is a life-threatening organ dysfunction due to dysregulated immune responses against infection. It affects approximately 50 million patients worldwide annually, significantly impacting public health and health care costs; however, no specific therapeutics exist beyond supportive care^11^. During sepsis, PAMPs and DAMPs circulate the body and activate immune and non-immune cells, leading to the production of proinflammatory mediators, i.e. proinflammatory cytokines and DAMPs^4^. Dysregulated immune responses also include impaired bacterial phagocytosis/clearance and metabolic disruption^4,12^. These septic immune alterations cause disruption of the intestinal mucosal barrier and gut microbiota dysbiosis^2^. Bacterial translocation due to increased gut permeability has been considered one of the hallmarks of sepsis pathophysiology^2^. As connected by the portal vein, the liver can be affected directly by this pathogen infiltration via the gut. Dysbiosis, i.e. imbalance in gut microbiota, also contributes to liver injury in sepsis as balanced gut nutrients and physiological levels of microbial products are essential for maintaining liver homeostasis^13^. The importance of understanding the gut–liver axis in sepsis is supported by the high mortality of septic patients with liver injury, which is reported to be even higher than that of lung injury, a well-known lethal complication of sepsis^14^. One study demonstrated that in sepsis, the translocation of gut microbial components attenuated liver stearoyl CoA desaturase-1 (SCD-1) activity, a key enzyme in hepatic de novo lipogenesis, resulting in compromised plasma membrane fluidity of red blood cells and dysregulated iron recycling^15^. We have recently unveiled a novel mechanism of gut–liver crosstalk in sepsis facilitated by gut-primed neutrophils^7^. Neutrophils interact with gut IELs, migrate to the liver and activate Kupffer cells by releasing neutrophil extracellular traps (NETs), exacerbating sepsis-induced acute liver injury (Fig. 1).

### Intestinal ischaemia–reperfusion injury

Acute mesenteric ischaemia is a devastating clinical entity caused by embolism, thrombosis or systemic hypotension, as well as in iatrogenic insults such as solid organ transplant. Even after resolution of the offending pathology, the tissue insult is continued by reperfusion injury. Acute mesenteric ischaemia is reported to have >50% mortality, even recognized and treated within 24 h after the onset^16^. Initial ischaemia leads to hypoxia, which severely affects the intestinal tissues by inducing cellular stress and cell death, including necrosis. The primary focus against intestinal ischaemia is to reestablish the blood flow to minimize the injury within the corresponding vascular territory^16^. However, even if the ischaemia is resolved, the subsequent reperfusion allows the systemic exposure of inflammatory mediators that are released by stressed or dead cells. For instance, a DAMP, eCIRP, is released from damaged cells and exacerbates gut I/R injury by inducing aberrant immune responses^16^. Reperfusion leads to increased capillary permeability and a breakdown of the intestinal barrier. In addition to the release of cellular compounds, gut contents also enter the body owing to a compromised intestinal barrier resulting from ischaemic damage, thus leading to a dysregulated immune response to the invading pathogens and PAMPs, a state called enterogenic sepsis^17^. The liver is particularly susceptible to this systemic inflammation and intestinal I/R often causes liver injury. Elevated levels of the liver enzymes, AST and ALT, are routinely observed following gut I/R^18^. Signalling molecules involved in gut I/R-induced liver injury include NF-κB, Nrf2/ARE and AMPK/SIRT1^18^. It has been reported that HMGB1-associated necroptosis of the liver tissue and M1 polarization of Kupffer cells contribute to gut I/R-induced liver injury as well^19^.

### Acute alcoholic hepatitis

Alcohol misuse can cause a variety of liver injuries, namely, alcohol-associated liver diseases. Symptomatic steatohepatitis due to alcohol misuse is called alcoholic hepatitis^20^. Patients with alcoholic hepatitis have a high mortality rate, which is reported to be 17–50%^20^. Toxic metabolites of ethanol can induce hepatocyte damage by altering redox reactions and causing mitochondrial dysfunction^21^. Alcohol can also facilitate proinflammatory cell activation and recruitment^21^. The gut–liver crosstalk is considered important in the development of alcoholic hepatitis as alcohol consumption affects the gut environment to make the liver more vulnerable to inflammation. Alcohol consumption can cause significant changes in gut microbial composition and induce intestinal-barrier dysfunction, exacerbating alcoholic hepatitis through the gut–liver axis^3^. Based on these mechanisms, several therapeutic approaches have been investigated in the setting of alcoholic hepatitis. Sterilizing the gut with antibiotics, targeting endotoxaemia by bovine colostrum and hyperimmunized bovine colostrum IMM-124E, and modulating gut microbiome by *Lactobacillus* and faecal transplantation are all under clinical trials for alcoholic hepatitis. The active clinical trials highlight the significance and clinical relevance of gut–liver crosstalk in this inflammatory disorder^20^.

### Drug-induced acute liver injury

Drug-induced liver injury is a severe prevalent disorder resulting from an adverse reaction to drugs. In the West, drug-induced liver injury is the leading cause of acute liver failure^22^. Acetaminophen overdose is the most common cause of acute liver failure, but it can also arise from a wide range of drugs, including antibiotics, lipid-lowering agents and antihypertensive drugs^22^. Drugs can disrupt the balance of the gut microbiome by altering its metabolic processes or structural integrity, compromising the intestinal barrier. For example, antibiotics can rapidly reduce microbial diversity, deplete beneficial commensals and promote the expansion of pathobionts^23^. Proton pump inhibitors (PPIs) can increase oral taxa and potential pathogenic bacteria in the microbiome. These changes have been linked to *Clostridioides difficile* infections^24^. Non-steroidal anti-inflammatory drugs (NSAIDs) can disrupt mucosal defences and cause gut erosions and ulcers — so‑called “NSAID enteropathy”^25^. Moreover, PPIs can further increase the severity of NSAID enteropathy by inducing dysbiosis^26^. As a result of drug-induced intestinal damage, the translocation of bacterial products can induce the activation of pattern recognition receptors (PRRs) in the liver, causing hepatic inflammation^27^. Maintaining the physiological microbiome by probiotics, including *Akkermansia muciniphila* and *Lactobacillus* species, is known to be protective against drug-induced liver injury, such as acetaminophen-induced hepatic failure^27^.

### Viral hepatitis

Acute viral hepatitis primarily arises from hepatotropic viruses, such as hepatitis A, B, C and E viruses. Hepatitis A and E viruses are transmitted through the oral faecal routes, implying their effects on gut microenvironment^28,29^. Viral exposure or shedding within the intestinal lumen can disrupt gut flora, influence barrier integrity and facilitate microbial or PAMP translocation into the portal circulation. Interestingly, prolonged dysbiosis was observed in patients with hepatitis A and E infections^28,29^. It has been reported that the microbiota also plays a role in hepatitis B virus-related acute-on-chronic liver failure (HBV-ACLF). Significant differences were observed in the microbiome diversity between patients with HBV-ACLF and HBV-healthy controls^30^. Microbiota composition was proposed as a progression marker as *Enterococcus* was enriched in the progression group whereas *Faecalibacterium* was enriched in the regression group in patients with HBV-ACLF^30^.

## Mechanisms of inflammatory gut–liver crosstalk

**Fig. 2:**
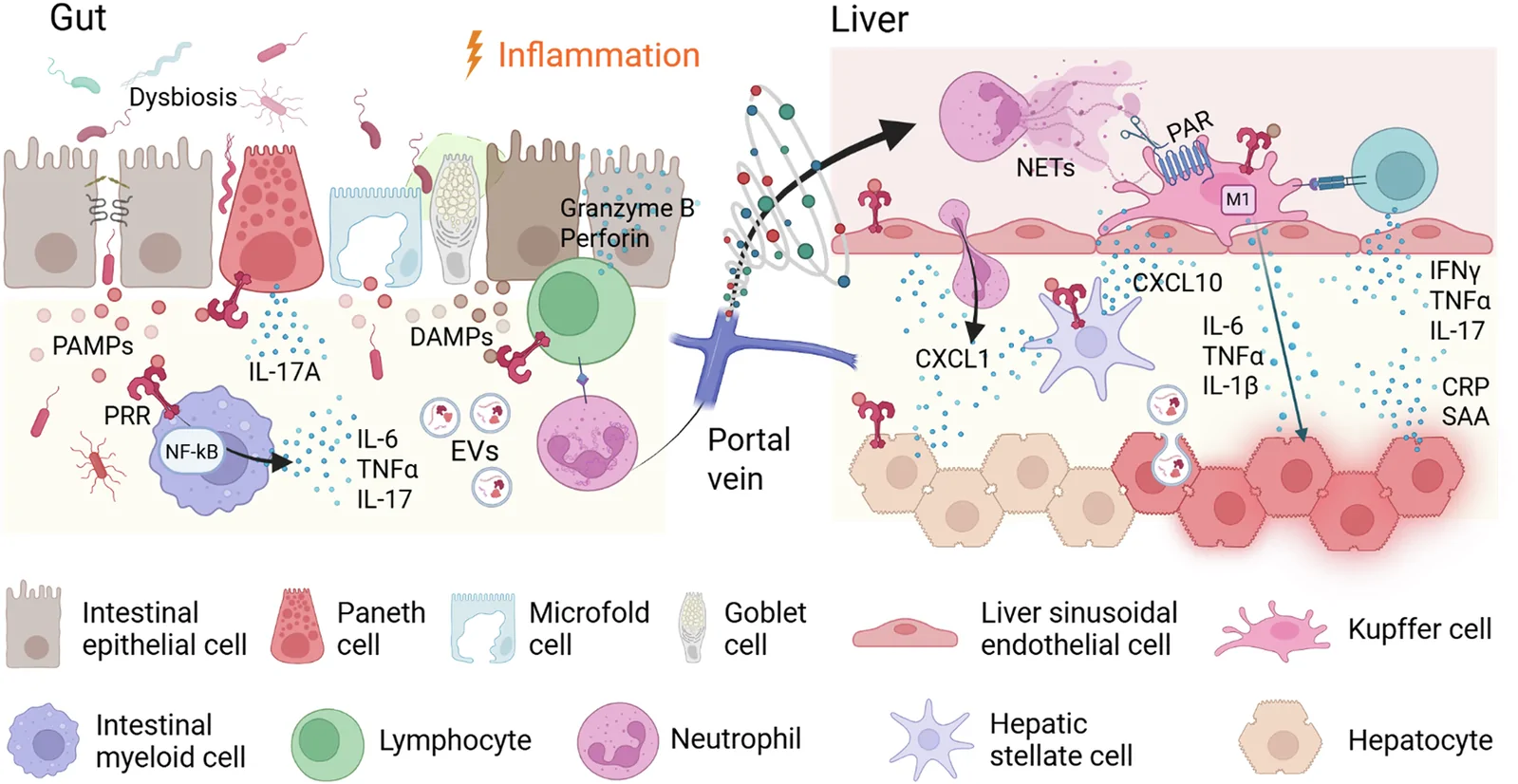
Mechanisms of gut–liver crosstalk in inflammation. During inflammation, dysbiosis increases gut-barrier permeability, leading to the translocation of pathogens and pathogen-associated molecular patterns (PAMPs). Damage-associated molecular patterns (DAMPs) can be released from stressed cells in the gut. Various cell types recognize PAMPs and DAMPs via pattern recognition receptors (PRRs) or interact with other cells, releasing cytokines, chemokines, cytotoxic granules and neutrophil extracellular traps (NETs). These proinflammatory mediators and activated cells translocate to the liver via the portal vein, activating various hepatic cells to produce additional proinflammatory mediators that exacerbate liver-tissue injury. CRP, C-reactive protein; EVs, extracellular vesicles; PAR, protease-activated receptor; SAA, serum amyloid A.

Gut–liver communication is mediated by the intestinal microbiome, microbial and cellular products, and immune cells^7,10^. Commensal microbiota modulate immune responses to maintain homeostasis, whereas dysbiosis and structural damage during acute insults increase portal translocation of microbial products, promoting hepatic inflammation^10^. PAMPs and DAMPs activate PRRs on immune and non-immune cells to exacerbate inflammation along the gut–liver axis^3^. Gut and liver resident cells and circulating immune cells contribute to gut–liver crosstalk by producing proinflammatory mediators or migrating from the gut to the liver^7^. Relevant mechanisms for gut–liver crosstalk in acute inflammation are mentioned, even though some supporting studies were conducted in subacute or chronic conditions (Fig. 2).

### Microbiome

The gut microbiome is a diverse community of bacteria, archaea, fungi and viruses that maintain intestinal homeostasis and influence systemic physiology^10^. Commensal taxa produce metabolites including short-chain fatty acids (SCFAs) that support tight junction integrity, modulate mucosal immune responses and limit inflammation^1,10^. Disruption of the microbial community — dysbiosis — can result from acute insults such as I/R, sepsis or trauma^13^. Dysbiosis often involves loss of beneficial commensal organisms and expansion of opportunistic species, impairing barrier function and facilitating the translocation of microbial products into the portal circulation^1^.

Several studies highlight the role of the microbiome in acute liver injury. In a mouse model of hepatic I/R, reduction of gut microbiota with antibiotics reduced liver damage, whereas faecal–microbiota transplantation restored susceptibility, demonstrating the pathogenic role of the microbiome^31^. *Enterococcus faecalis*, a common commensal bacterium in the human gut microbiome, can secrete cytolysin, which causes hepatocyte death and liver injury, and the presence of cytolysin-positive *Enterococcus faecalis* correlates with hepatic dysfunction and mortality in patients with alcoholic hepatitis^32^. Pretreatment of liver I/R mice with antibiotics reshaped the gut microbiota, especially decreasing the relative abundance of Firmicutes and increasing the relative abundance of *Clostridium*, and attenuated hepatic injury by alleviating cell apoptosis, reducing the inflammatory response and decreasing the recruitment of CCR2^+^ monocytes^33^. In a rat hepatic I/R model, administration of *Clostridium butyricum* improved hepatic outcomes by lowering serum ALT/AST, reducing LPS levels and restoring SCFA profiles^34^.

### Pathogen-associated molecular patterns

PAMPs are molecular structures derived from microorganisms, such as bacteria, viruses and fungi, that can trigger immune responses through PRRs. When the gut barrier is compromised, PAMPs can enter the circulation to reach the liver via the portal vein^1^. Gut-derived PAMPs activate PRRs, including Toll-like receptors (TLRs) and NOD-like receptors (NLRs). These are expressed on liver cells, such as Kupffer cells, hepatocytes, endothelial cells and stellate cells, and when activated induce the production of proinflammatory mediators, thereby facilitating gut–liver crosstalk^35–38^. LPS, specifically lipid A, is the major PAMP present on the outer membrane of Gram-negative bacteria, e.g. Enterobacteriaceae, *Bacteroides*. During acute insults, such as sepsis, I/R and trauma, tight junctions and the mucosa are damaged, allowing paracellular or transcellular LPS passage^2^. Portal LPS activates hepatic TLR4 on Kupffer and sinusoidal endothelial cells, directly causing liver tissue injury^38^. LPS can also activate TLRs on intestinal macrophages, inducing the release of proinflammatory cytokines such as TNF and IL-1β^39^. These cytokines amplify local inflammation to disrupt the epithelial barrier and can also reach the liver to directly promote hepatic inflammation and injury^40^.

In addition to LPS, other microbial-derived PAMPs contribute to gut–liver signalling. Peptidoglycan (PGN) released from the wall of commensal Gram-positive and Gram-negative bacteria can be sensed by intracellular receptors such as NOD1 and NOD2, leading to NF-κB activation and proinflammatory responses in liver tissues^41^. Circulating bacterial DNA — specifically unmethylated CpG DNA motifs — is also elevated in settings of gut-barrier disruption and recognized by TLR9 in hepatic cells, thereby linking microbial translocation to liver inflammation^42^. Moreover, flagellin, the structural protein of bacterial flagella, has been shown to activate TLR5 to induce acute liver inflammation and injury in animal models^43^.

### Damage-associated molecular patterns

DAMPs are endogenous molecules released from stressed or damaged cells that induce immune responses via PRRs^4^. eCIRP, an RNA chaperone intracellularly, can be secreted via gasdermin D pores and exosomes or released through the process of necroptosis, an MLKL-dependent programmed cell death^44^. eCIRP is elevated in inflammatory conditions such as intra-abdominal infection and gut I/R^16,45^. Following gut inflammation, eCIRP activates TLR4 on Kupffer cells to induce their M1 polarization and cytokine release, contributing to liver injury^46^. HMGB1 is a nuclear protein serving as a DAMP extracellularly. During cellular injury, HMGB1 translocates from the nucleus to the cytoplasm and is released either passively from necrotic cells or actively through secretory lysosomes^47^. HMGB1 acts on TLR4 and the receptor for advanced glycation end products (RAGE), both of which amplify proinflammatory signalling and cytokine release^47^. Following gut I/R, HMGB1 induces Kupffer cell M1 polarization and liver injury^19^. Mitochondrial-derived DAMPs, including mitochondrial DNA and N-formyl peptides, released from damaged intestinal cells, can activate TLR9 or cGAS–STING signalling in Kupffer cells, triggering type I interferon and IL-1β production^48,49^.

## Cellular contributors in inflammatory gut–liver crosstalk

The gut–liver axis depends on coordinated activities among intestinal, hepatic and circulating cells. In the gut, epithelial cells and myeloid cells regulate barrier integrity and microbial balance but are prone to aberrant activation during inflammation^9,50^. Hepatic cells, including Kupffer cells, sinusoidal endothelial cells (LSECs), hepatic stellate cells (HSCs) and hepatocytes, sense and respond to gut-derived signals^51–54^. Circulating immune cells, particularly lymphocytes and neutrophils, dynamically connect the two organs — migrating from inflamed gut tissue through the portal vein to the liver, where they amplify inflammation and tissue injury^7,55^. A transcriptomic study of liver tissue from intra-abdominal sepsis using single-cell RNA sequencing revealed increased adhesion factors in LSECs, lymphocyte dysfunction, macrophage activation and neutrophil recruitment with subsequent NETosis^56^.

### Neutrophils

Neutrophils are the predominant leukocytes in the human blood and the initial drivers of acute inflammation owing to their role in the innate immune response^57^. When inflammation occurs in the gut, neutrophils swiftly migrate to the epicentre following chemokines^58^. Besides eliminating pathogens, neutrophils can also be activated by inflammatory mediators or by interacting with activated cells in the gut. Neutrophils have increased lifespan during inflammation and via reverse transendothelial migration they return back to the circulation^59^. After coming out of the gut, they migrate through the portal vein to reach the liver. Neutrophils release a variety of proinflammatory molecules that can cause liver tissue injury. NETs are web-like structures composed of DNA, histones and microbicidal proteins released from neutrophils primarily to eliminate pathogens^4^. Abundance of NETs can be detrimental to tissues, and NETs are known to contribute to liver injury. Our laboratory has recently demonstrated a dynamic mechanism that CD112^+^ neutrophils infiltrate into the inflamed gut and, primed by IELs, subsequently migrate to the liver via the portal vein, and activate Kupffer cells to cause hepatic injury^7^. We have identified that these neutrophils produce excessive NETs containing proteases such as neutrophil elastase, which activates protease-activated receptor 1 on Kupffer cells, inducing M1 polarization and cytokine production with downstream liver injury^7^.

### Intestinal epithelial cells

Intestinal epithelial cells (IECs) play a pivotal role in maintaining homeostasis, not only for the gut but also for the entire body. IECs form a barrier in the intestinal tract to modulate nutrient absorption and pathogen exclusion^60^. Tight-junction proteins, such as claudins, occludin, junctional adhesion molecules and zonula occludens, are essential for maintaining IEC barrier integrity. These proteins assemble into multiprotein complexes that constitute a continuous paracellular seal at the apical junctions^2^. During acute inflammation, proinflammatory cytokines can alter the expression and localization of tight-junction proteins via the activation of myosin light-chain kinase^2^. These changes increase paracellular permeability by promoting tight junction disassembly and reorganization, leading to compromised epithelial barrier integrity. This allows the invasion of intestinal pathogens and related molecules, such as LPS, to cause the systemic exposure of these inflammatory components^9,60^. Moreover, IECs express PRRs, including TLR4, and can sense PAMPs and DAMPs. This will cause IELs to release proinflammatory mediators into the local environment as well as the systemic circulation^61^. The intestinal barrier defects and increased production of proinflammatory molecules heavily affect the liver owing to the close anatomical connection by the portal vein.

### Paneth cells

Paneth cells are specialized secretory epithelial cells found in the crypts in the small intestine. They contribute to intestinal and systemic homeostasis by producing antimicrobial peptides, such as α-defensins, lysozyme and secretory phospholipase A2. Together, these help to regulate the intestinal microbiota and prevent bacterial overgrowth and translocation^62^. Loss or dysfunction of Paneth cells impairs the mucosal barrier, leading to microbial imbalance and the leakage of bacterial products (PAMPs) into the portal circulation, thereby promoting hepatic inflammation. An antimicrobial peptide α-defensin deficiency in Paneth cells is reported to exaggerate bacterial translocation and hepatic inflammation^63^. Beyond antimicrobial defence, Paneth cells also play a role in regulating portal hypertension in response to microbial signals^64^. Moreover, Paneth cells express PRRs and respond to microbial or inflammatory stimuli by releasing cytokines that influence distant organs. A study has shown that Paneth cells release a proinflammatory cytokine IL-17A, which can exacerbate liver injury after hepatic I/R^65^.

### Goblet cells

Goblet cells are a subset of intestinal epithelial cells that secrete mucins, such as the gel-forming MUC2, and complementary mediators, thereby supporting barrier integrity. Upon encountering pathogens or stimulation by proinflammatory mediators, goblet cells increase mucus secretion to protect against infection^66^. However, these stimuli can also impair the mucus layer, potentially permitting pathogen translocation into the portal circulation, as mucus-barrier regulation is highly complex and mucin expression, processing and mucus properties are dynamically regulated by host and external factors (e.g. pathogens, diet, antibiotics)^66^. In addition to forming the mucus barrier, goblet cells sample and deliver luminal antigens to antigen-presenting cells in the lamina propria through goblet-cell-associated antigen passages. A recent study has shown that alcohol consumption downregulates muscarinic acetylcholine receptor M4 (mAChR4) to reduce goblet-cell-associated antigen passage formation, disrupting antimicrobial immunity^67^. Goblet-cell-specific activation of mAChR4 was essential and sufficient to prevent ethanol-induced steatohepatitis^67^, highlighting the importance of this mechanism within the gut–liver axis.

### Microfold cells

Microfold cells are epithelial cells that reside in the follicle‑associated epithelium overlying Peyer’s patches. They are derived from intestinal stem cells in response to proinflammatory mediators, including TNF, via the activation of receptor activator of NF-κB ligand (RANKL)/SPIB pathway^68^. Microfold cells mediate transcytosis of luminal contents across the epithelium to underlying antigen‑presenting cells. Microfold-cell-driven antigen sampling fosters secretory IgA production and intestinal tolerance, maintaining barrier integrity^69^. However, their transport capacity can also be exploited by pathogens to invade the host. Indeed, some enteric pathogens, such as *Salmonella* Typhimurium, preferentially target microfold cells to traverse the epithelium, underscoring their dual role in immune surveillance and vulnerability^70^. In this regard, microfold cells may act as a critical checkpoint in gut–liver crosstalk during acute inflammation, even though direct evidence remains limited. Inflammatory and microbial signals can induce microfold-cell expansion and heightened activity, enhancing host defence but also increasing hepatic exposure to luminal antigens and pathogens via the portal vein when mucosal containment fails.

### Intestinal myeloid cells

Intestinal myeloid cells, i.e. macrophages and dendritic cells, mainly reside in lamina propria and maintain mucosal homeostasis by sampling antigens, clearing apoptotic or damaged cells, and producing regulatory cytokines^71,72^. They express PRRs and can sense PAMPs and DAMPs, which can induce proinflammatory signalling, including NF-κB activation^50^. During gut inflammation, these myeloid cells can shift towards proinflammatory phenotypes, releasing cytokines and chemokines to increase intestinal permeability and promote microbial translocation^50^. As a result, proinflammatory mediators or pathogens enter the portal circulation and reach the liver. In the liver, hepatic cells sense these myeloid cell-derived signals and respond by producing further proinflammatory cytokines and chemokines, as well as reactive oxygen species, exacerbating hepatic inflammation^54^. In patients with liver disease, activated CD14^+^TREM-1^+^iNOS^+^ intestinal macrophages were observed along with increased intestinal permeability^5^. These macrophages release proinflammatory cytokines and chemokines, including IL-6, IL-8, CCL2 and CCL13^5^. A recent study has shown that dysfunction of intestinal blood-vessel-associated macrophages, which failed to adequately line submucosal blood vessels, was found in mice with liver disease^73^. Some intestinal myeloid populations may migrate and directly influence liver outcomes. In a colitis model, macrophages engulfed insoluble indirubin within Peyer’s patch and transported it to the liver, leading to hepatic injury^6^.

### Kupffer cells

Kupffer cells are the resident macrophages in the liver residing in the sinusoids, the specialized capillaries that facilitate molecular exchange between the blood and hepatocytes. As professional phagocytes, Kupffer cells maintain liver homeostasis by clearing pathogens, endotoxins, apoptotic cells and other toxic substances from the portal circulation^74^. Kupffer cells play a major role in gut–liver crosstalk as they are highly sensitive to proinflammatory molecules migrating from the gut and can produce an abundance of proinflammatory mediators in response to these stimuli^8^. When gut integrity is compromised, microbial products, i.e. PAMPs, enter the bloodstream. In addition, DAMPs are released from stressed or damaged cells in the gut in response to inflammation. These molecules are transported to the liver via the portal vein to activate Kupffer cells through PRRs, including TLRs^51^. PRR stimulation on Kupffer cells activates its downstream signalling pathway, such as NF-κB and MAPK^75^. Macrophages in general are known to consist of distinct phenotypes, i.e. M1 and M2. PAMPs and DAMPs can induce M1 polarization of Kupffer cells via PRR activation. M1 Kupffer cells release proinflammatory cytokines, e.g. TNF, IL-6, IL-1β, as well as reactive oxygen species and nitric oxide^7,46^. These Kupffer cell products induce hepatocyte damage and the activation of other hepatic cells, such as stellate cells, as well as recruiting other immune cells to exacerbate liver injury^51^.

### Liver sinusoidal endothelial cells

LSECs form the highly specialized endothelium lining the hepatic sinusoids and serve as the first parenchymal cells exposed to portal venous blood containing gut‑derived products, such as microbial molecules, highlighting the importance of this cell type in the gut–liver axis^76^. LSECs not only form a barrier within the hepatic sinus but also play an important role in regulating hepatic vascular pressure and inflammation^77^. Therefore, LSEC dysfunction is characterized by multiple pathological phenomena, including the loss of fenestrae, inflammatory activation and the acquisition of vasoconstrictive and prothrombotic functions. LSECs express PRRs, allowing them to respond to gut‑derived products, such as LPS, by producing proinflammatory mediators when gut integrity is compromised or microbial translocation increases^52^. They can also upregulate adhesion molecules and secrete chemokines, facilitating recruitment of immune cells, which amplify inflammation^76^. In a colitis model, LSEC damage has been shown to contribute to liver injury by inducing hepatic CXCL1 expression and neutrophil recruitment^52^.

### Hepatic stellate cells

HSCs reside in the subendothelial space of Disse, interposed between LSECs and hepatocytes. They store vitamin A, regulate extracellular matrix turnover and maintain sinusoidal homeostasis^78^. During inflammation, PAMPs and DAMPs can activate PRRs, such as TLR4, on HSCs, resulting in liver-tissue injury. It has been shown that the activation of MyD88, a critical adaptor protein for TLR signalling, in HSCs increased the secretion of CXCL10, which promoted macrophage M1 polarization through CXCR3^79^. In the context of gut–liver crosstalk, HSCs can be influenced by gut‑derived stimuli, such as bacterial metabolites, which reach the liver owing to increased gut permeability and activate PRRs on HSCs^78^. HSCs respond to microbiota or low-level LPS by producing CXCL1 in a TLR4-dependent manner^80^. The involvement of HSCs in the gut–liver axis is also supported by a study showing that HSCs were activated after colitis, as indicated by upregulated α-SMA, a marker of HSC activation associated with fibrosis^81^. Another study has shown that gut microbiota metabolite 3-Indolepropionic acid can directly activate HSCs through reactive oxygen species-induced JNK and p38 signalling pathways^53^.

### Hepatocytes

Hepatocytes constitute approximately 80% of the liver’s mass and are the primary parenchymal cells responsible for maintaining systemic homeostasis by regulating metabolism and synthesizing plasma proteins, such as albumin and coagulation factors^82^. Hepatocytes are not merely passive downstream targets of the inflammatory cascade; they can directly sense and respond to gut-derived signals. They express a wide array of PRRs, including TLRs and NLRs, enabling them to detect PAMPs and DAMPs from the gut^35,54^. Activated hepatocytes produce inflammatory cytokines and acute phase proteins, such as C-reactive protein and serum amyloid A, amplifying local and systemic inflammation^83^. A study has shown that gut-derived LPS induced CXCL1 expression in hepatocytes through a TLR4-dependent mechanism, leading to an accumulation of CXCR2^+^ polymorphonuclear myeloid-derived suppressor cells (PMN-MDSCs)^84^. Gut-derived IL-13, a critical mediator of allergic inflammation, has also been shown to directly activate hepatocytes via the IL-13R/JAK2/STAT6 axis^85^. Gut-microbiota-derived tryptophan metabolites attenuate inflammatory responses in hepatocytes via the aryl hydrocarbon receptor, whereas these metabolites are depleted by a high-fat diet, indicating a potential role in steatohepatitis^86^.

### Lymphocytes

Lymphocytes play pivotal roles in both the adaptive and innate-like immune systems, functioning as key mediators of antigen recognition, immune regulation and tissue homeostasis. They consist of T cells, B cells and innate-like lymphocytes such as mucosal-associated invariant T (MAIT) cells, natural killer T (NKT) cells, γδ T cells and innate lymphoid cells (ILCs), each contributing uniquely to host defence and immune regulation^87^. In the gut, lymphocytes respond dynamically to microbial and dietary antigens. Activated CD4^+^ and CD8^+^ T cells, as well as innate-like subsets, can release proinflammatory cytokines such as IFNγ and IL-17, which enhance epithelial permeability and enable translocation of microbial products to the portal circulation — indirectly affecting the liver^88,89^. A study has shown that IELs can be activated by eCIRP during intra-abdominal bacterial infection and produce cytotoxic granules, granzyme B and perforin, which can exacerbate gut-tissue injury, further increasing intestinal permeability^90^. Moreover, gut-derived lymphocytes expressing specific homing receptors, including α_4_β_7_ integrin, can reach the liver when hepatic endothelium expresses the corresponding ligand MAdCAM-1 during inflammation, exacerbating liver injury^55^. The modulation of microbiota can promote ILC3 homing to the liver via the CXCL16/CXCR6 axis, where hepatic ILC3s regulate inflammation and tissue repair through IL‑22^91^. Within the liver, resident and recruited lymphocytes act as sentinels that continuously sample gut-derived microbial and metabolic products delivered via the portal vein. Hepatic MAIT cells, NKT cells and γδ T cells, enriched within the sinusoids, can be activated by bacterial metabolites or lipid antigens presented by phagocytes and produce IL-17, IFNγ and TNF, promoting inflammation^87^.

### Extracellular vesicles

Extracellular vesicles (EVs) — including exosomes and microvesicles — are nanosized particles released by various cell types, including bacteria. They carry lipids, nucleic acids and proteins, facilitating cellular communication by transporting these molecules^92^. EVs can circulate dynamically throughout the body, thereby contributing to organ-to-organ crosstalk, including the gut–liver axis. For example, EVs released from IECs have been shown to promote liver injury during acute alcoholic hepatitis by reducing hepatocyte viability and increasing lipid accumulation^93^. The function of EVs is dependent on their cargo. miR-21a-5p contained in IEC EVs attenuates hepatic inflammation by inhibiting macrophage M1 polarization, whereas miR-145a-5p promotes hepatic lipid accumulation via suppressing BTG1^94^. EVs derived from breast milk contain microRNAs and proteins associated with intestinal-barrier function and protect intestinal-barrier integrity in the gut–liver axis^95^. Gut microbial DNA-containing EVs from obese hosts activate the cGAS/STING pathway in hepatocytes, enhancing inflammatory reactions^92^.

## Liver-to-gut axis in acute inflammation

The liver-to-gut arm of the gut–liver axis provides a route by which hepatic stress and inflammation reshape intestinal physiology, immunity and microbial ecology^1,2^. Anatomically, the liver and gut are connected via the biliary tract, enabling direct delivery of liver-derived factors into the intestinal lumen. Bile acids (BAs) are key liver-derived signalling molecules that act directly on the gut. Beyond their classical role in lipid digestion, BAs act as signalling molecules that regulate epithelial integrity, antimicrobial defence and mucosal immune tone through receptors such as FXR and TGR5^1,2^. During acute inflammation, hepatic injury alters BA synthesis, conjugation and transport, yielding a remodelled BA pool enriched in more hydrophobic and potentially cytotoxic species^2^. The liver also regulates the intestinal environment through biliary secretion of immune effectors. Secretory IgA, transported into bile and delivered to the intestinal lumen, plays a critical role in immune exclusion by limiting microbial adherence to the epithelium. Acute liver dysfunction can disrupt IgA production or transport, weakening this protective barrier and allowing increased microbial–epithelial interactions^96^. These biliary changes can impair epithelial-cell membranes, disrupt tight junctions and promote mucosal inflammation, while also driving the gut microbiota towards a dysbiotic state. In parallel with the biliary route, the liver communicates with the gut through the systemic circulation^2^. Cytokines, acute-phase proteins and complement components produced by hepatocytes and non-parenchymal cells during acute inflammatory responses act downstream on intestinal epithelial and immune cells. These signals alter epithelial turnover, suppress tight-junction protein expression and impair mucus-layer integrity, thereby increasing intestinal permeability and reinforcing an inflammatory feedback loop within the gut–liver axis^2^.

## Therapeutic approaches targeting inflammatory gut–liver crosstalk

**Fig. 3:**
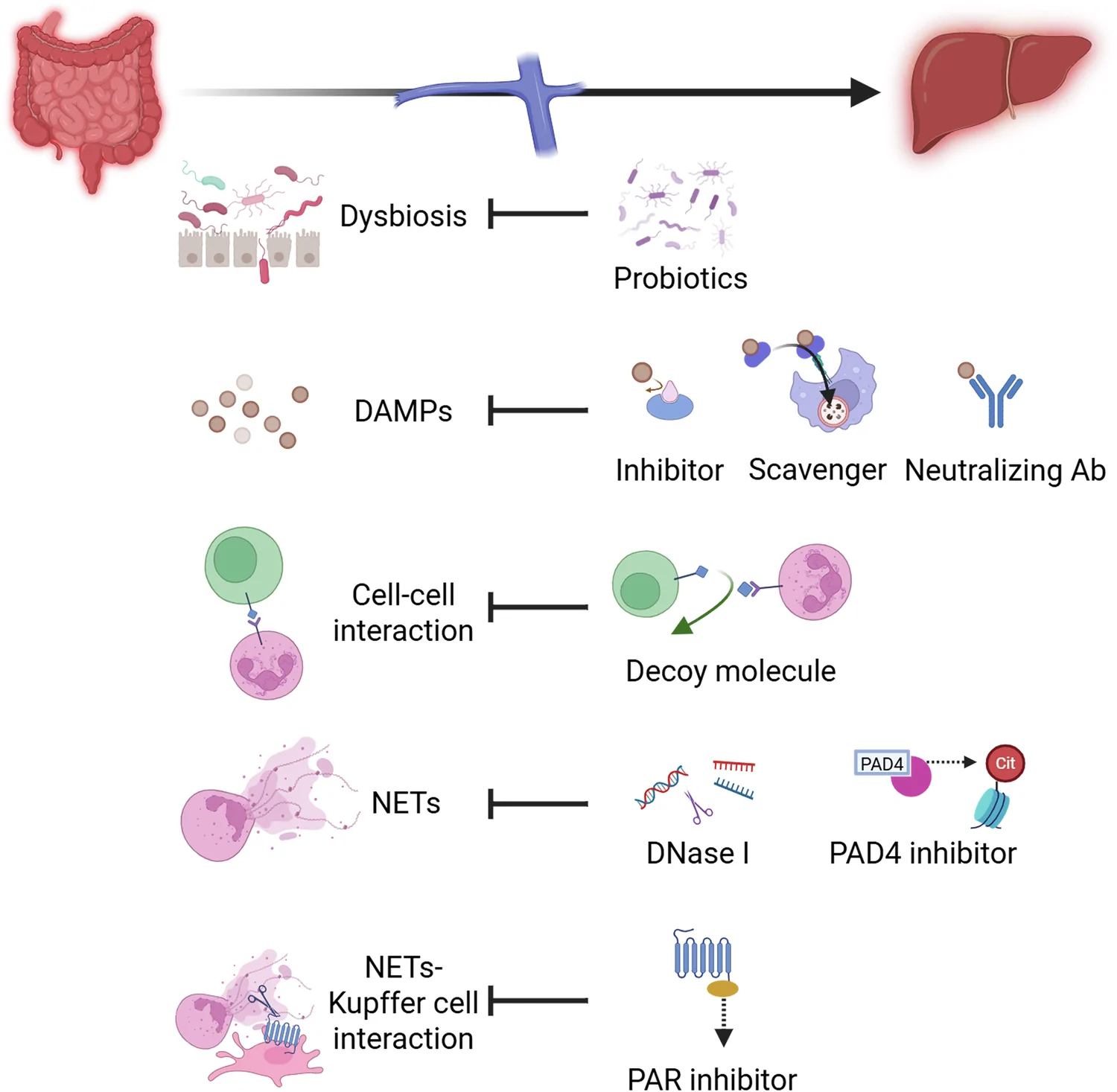
Therapeutics for inflammatory gut–liver crosstalk. Therapeutic strategies targeting the gut–liver axis involve reversing dysbiosis with probiotics and pharmacologically inhibiting proinflammatory molecules, signalling pathways and cellular interactions. Ab, antibody; DAMP, damage-associated molecular pattern; NET, neutrophil extracellular trap; PAD4, peptidylarginine deiminase 4; PAR, protease-activated receptor.

Therapies targeting the gut–liver axis during systemic inflammation are aimed at reducing intestinal-barrier disruption, microbial translocation and hepatic immune activation. Strategies would include reinforcing intestinal-barrier integrity and modulating the microbiota. Several preclinical studies illustrate these concepts. In drug-induced liver-injury models, probiotics and postbiotics reduced intestinal permeability, decreased portal blood LPS and mitigated hepatic inflammation^27^. In an I/R liver-injury model, preconditioning of the microbiome with antibiotics attenuated hepatic injury by macrophage metabolic reprogramming^31^. However, translating microbiota-targeted approaches to the clinical acute setting is challenging, as most patients in the intensive care unit cannot undergo preconditioning or gradual microbiome modulation and are often unable to tolerate enteral feeding in the very acute phase owing to unstable vital signs or severe intestinal injury, including perforation^97^. Therefore, strategies that directly protect the intestinal barrier or inhibit gut/hepatic immune activation may be more practical as acute interventions. Targeting eCIRP using small peptides or its scavenging molecule mitigates gut I/R by reducing systemic inflammation and maintaining gut-barrier function, implicating a possible direction of improving liver function following gut I/R injury^16,98^. NETs can be another potential therapeutic target as they activate Kupffer cells and contribute to liver injury during acute inflammation^7^. NET-targeted therapies, including DNase I and peptidylarginine deiminase 4 (PAD4) inhibitors, are in clinical trials for inflammatory conditions^99^. We have recently developed a 13-amino acid peptide derived from CD112 to inhibit proinflammatory neutrophil-IEL interaction in the gut^100^. This peptide has been shown to inhibit NETosis induced by IELs and protect mice from hepatic injury after gut I/R and sepsis^100^. These recent studies suggest a potential therapeutic approach to inflammatory gut–liver crosstalk beyond microbiome modulation (Fig. 3).

## Conclusions and future perspectives

The gut–liver axis represents a tightly integrated system that maintains immune and metabolic homeostasis but becomes a major source of inflammatory amplification during acute insults. Intestinaltissue injury, including barrier disruption and dysbiosis, allows microbial and inflammatory mediators, such as PAMPs and DAMPs, to enter the portal circulation, activating hepatic immune and parenchymal cells, thereby propagating injury. Diverse cell types — including intestinal epithelial and myeloid cells, Kupffer cells, LSECs, HSCs and lymphocytes — contribute to interorgan communication through cytokines, chemokines and metabolic signals. Therapeutic modulation of the gut microbiome or intestinal barrier demonstrates potential in preclinical models, yet remains difficult to implement in critically ill patients. Strategies that can target cellular responses during inflammation may have translational potential to overcome this limitation.

Although recent studies have begun to dissect cellular interactions within the gut–liver axis, the spatiotemporal dynamics of gut-derived signals in acute inflammation remain incompletely understood. High-resolution methods such as spatial transcriptomics and in vivo cell tracking will be crucial to delineate how gut and liver immune networks interact in real time during acute inflammation.
